# Consequence of Synthetic Bone Substitute Used for Alveolar Cleft Graft Reconstruction (Preliminary Clinical Study)

**DOI:** 10.1055/a-2113-3084

**Published:** 2023-08-31

**Authors:** Rawaa Y. Al-Rawee, Bashar Abdul-Ghani Tawfeeq, Ahmed Mothafar Hamodat, Zaid Salim Tawfek

**Affiliations:** 1 Department of Oral and Maxillofacial Surgery, Al-Salam Teaching Hospital. Mosul, Iraq; 2 Paedo Ortho Prevention Department, Alnoor University College, Mosul, Iraq; 3 Department of Pediatric Surgery, Al-Khansa Teaching Hospital, Mosul, Iraq; 4 Department of Oral and Maxillofacial Surgery, Al-Noor University College, Mosul, Iraq

**Keywords:** alveolus grafting, synthetic bone substitute, secondary bone grafting, alveolar cleft, reconstruction

## Abstract

**Background** The outcome of alveolar grafting with synthetic bone substitute (Osteon III) in various bone defect volumes is highlighted.

**Methods** A prospective study was accomplished on 55 patients (6–13 years of age) with unilateral alveolar bone cleft. Osteon III, consisting of hydroxyapatite and tricalcium phosphate, is used to reconstruct the defect. Alveolus defect diameter was calculated before surgery (V1), after 3 months (V2), and finally after 6 months (V3) postsurgery. In the *t* -test, a significant difference and correlation between V1, V2, and V3 are stated. A *p-* value of 0.01 is considered a significant difference between parameters.

**Results** The degree of cleft is divided into three categories: small (9 cases), medium (20 patients), and large (26 cases).The bone volume of the clefted site is divided into three steps: volume 1: (mean 18.1091 mm ^3^ ); step 2: after 3 months, volume 2 resembles the amount of unhealed defect (mean 0.5109 mm ^3^ ); and the final bone volume assessment is made after 6 months (22.5455 mm ^3^ ). Both show statistically significant differences in bone volume formation.

**Conclusion** An alloplastic bone substitute can also be used as a graft material because of its unlimited bone retrieval. Osteon III can be used to reconstruct the alveolar cleft smoothly and effectively.

## Introduction

Alveolar cleft (AC) is an anomaly resulting from a fusion discrepancy between maxillary and median nasal processes during fronto-nasal prominence growth; it is usually associated with cleft lip and palate congenital anomalies. 1 A common AC is situated in the area between canine and lateral incisors, affecting their eruption.

Von Eiselsberg was the first author to host the AC reconstruction using autologous tissue. 1 In 1908, Lexer used nonvascular bone graft materials for bone replacement. 2 Autologous bone materials involve the iliac crest, cranium, ribs, tibia, and mandible. 3

The timing of nasoalveolar fissure surgeries is divided into primary and secondary alveolus grafting according to the patient's age characteristic.

- Patients under the age of 2 are considered to be in the “primary stage.”
- Pre-adolescents and pre-teens, ages 2 to 5, are in the early secondary stage.
- Three secondary-stage patients, aged 5 to 16, are a mixed group.
- Patients over the age of 16 years fall into the category of “late secondary.” 4

In the 1970s, primary bone grafting (PBG) surgeries primarily used infant rib bone to treat ACs. 5 Avoiding serious complications such as anterior crossbite and retrusion of the midface are recorded concerning PBG. 5

“Secondary bone grafting (SBG) have been reported too; reconstruction of the AC is performed most frequently in the mixed dentition period (between 6 and 11 years).” 6 Numerous published articles highlight that SBG gives a good response to cleft reconstruction if correct, skilled surgical principles are followed. The study focused on transitional (conventional) secondary repair in patients aged 5 to 11 years. The most characteristic feature of this period is the presence of mixed dentition.

SBG acts mainly to prevent oro-nasal communication with food particle retention in the cleft area so that good oral hygiene is maintained and inflammation is diminished. Maxillary segment stabilization with facial aesthetic improvement can include tooth eruption and nasal support. 6

Many articles 7 8 highlight the controversy concerning the most appropriate material to select for alveolar bone reconstruction. Iliac crest autogenous bone graft is considered the gold standard approach for bone grafting because of its easy accessibility, comparatively abundant quality, and the ability to perform the simultaneous oral procedure.

It is well known that cancellous bone is a better grafting material because of the scaffold characteristic and vascularization in both osteoinduction and conduction. 9 On the other hand, there are also serious disadvantages, such as the harvesting of the graft from another region in the body with associated morbidity. For such reasons, bone grafting materials are available with diverse bridging effects, such as allogeneic freeze-dried bone, demineralized freeze-dried bone; demineralized bone matrix, and recombinant human bone morphogenetic protein. Reduced donor-site morbidity is the main criterion for using bone substitutes rather than autogenic materials to obtain good results. 10

Several pros are reflected upon the use of alloplastic material; as an example, donor site contribution from the patient is not needed, and subsequently any amount can be used according to defect size. 11 Hydroxyapatite (HA) is considered a synthetic bony mineral component, and furthermore, its osteoconductive substitute materials are used to fill the bony defect. “Synthetic calcium phosphate ceramics, with their excellent biocompatibility, can be considered as common alternatives to autogenous bone and xenograft materials.” 12

Genoss Company (Suwon, Korea) developed bony synthetic materials known as Osteon III substitutes. Osteon III materials have diverse particle size and shape and have different proportions of composition between HA and tricalcium phosphate (B-TCP). Osteon III is biphasic calcium phosphate. A HA surface coated with TCP (HA 60% + B-TCP 40%) can be used in the form of blocks, powder, or granules. Its particle size can be 0.5–1 mm or 1–2 mm, volume is 0.25–0.5 cc, and the volumetric porosity of Osteon III is approximately 77%. 12

The development of advanced technology such as cone beam computed tomography (CBCT) opens the surgical field to facilities for proper, accurate, and skilled surgeries with ideal follow-up of such cases to evaluate the bone bridging of the grafted material in the AC area pre- and postoperatively. In a study published by Shirota et al, it was stated that the amount of bone required for reconstructions can be estimated by surgical simulation software programs based on three-dimensional (3D) computed tomography images. 13

Soft tissue coverage of the grafted materials, whatever the source or origin of bony materials previously, was not so important, and little attention was paid to flap design. In 1981, several authors highlighted the extreme necessity for a good soft tissue closure of the grafted bone, and Abyholm et al were the first to focus on that. 14 Specific criteria should be maintained for good closure: complete covering with a mucoperiosteal flap without tension (watertight closure) for excellent surgical outcome.

### Aim of the Study

In light of the debates, the current research examines the success rate of AC grafting using bone substitutes of varying volumes. Also, it illustrates the change in bone volume and its relation to time after surgery. CBCT is an advanced technology that should be highlighted. The synthetic bone substitute Osteon III (Genoss) reaches its potential as a graft material.

## Methods

### Study Design

This is a prospective study examining the clinical outcome of 55 patients presented with AC repair performed at the Oral and Maxillofacial Surgery Unit of the Al-Salam Teaching Hospital, between January 2017 and 2021. Children were consecutive and with full consent of the parents.

#### Inclusion and Exclusion Criteria

Patients with unilateral AC cases not operated previously were included in this study, in addition patients aged between 6 and 13 years with no sex predilection as well as in regard to occlusion that does not affect graft stability were also included. Patients need added surgeries for the hard and soft palate at the same time with the alveolus cleft surgeries are excluded from the study. As well as participant who do not like to share, added to syndromic cleft type or medical disease presence are excluded from the study.

#### Ethical Approval

The study ethically was approved by the Human Ethical Scientific Approval Application Form for Research of the Nineveh Health Directory, Ministry of Health, Iraq with licensed number 67/120.

#### Sampling Method

Fifty-five patients selected according to inclusion criteria who have an AC and need bone grafting underwent surgeries under general anesthesia. Osteon III, consisting of HA and TCP, is used to reconstruct the defect. All patients have been seen by the Cleft Lip and Palate Committee authorized at Al-Salam Teaching Hospital. More than 100 patients per year attain the maxillofacial unit in Al-Salam Teaching Hospital with different congenital anomalies, including cleft lip and palate, various age groups, as well as numerous defects. Fifty-five patients are included in this study for surgical correction of their alveolar bone defect in the anterior region. The age range was 6 to 13 years old.

Patients pass through preoperative information, preparation, and approvals for reparation before surgery, including a demographic case sheet, examination, investigation, Multidisciplinary Team Clinic for Cleft Lip and Palate (MDT CLP) committee decision and agreement, ethical approval for publishing, and surgery approval from families. A written informed consent is discussed with the patient's family and signed before surgery; in addition, they approve the data use in this study.

#### Patient Examination

**Fig. 1:**
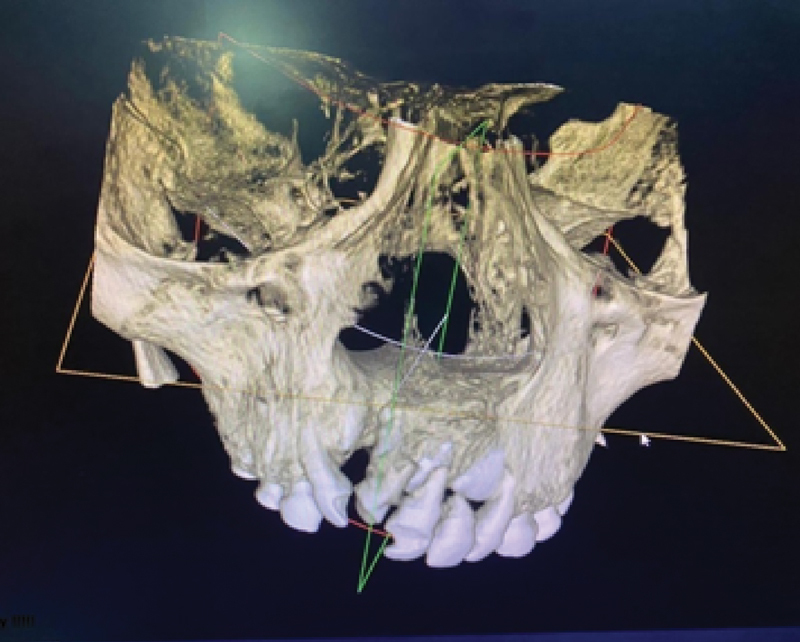
CBCT radiographic assessment of the clefted alveolar bone. CBCT, cone beam computerized tomography.

**Fig. 2:**
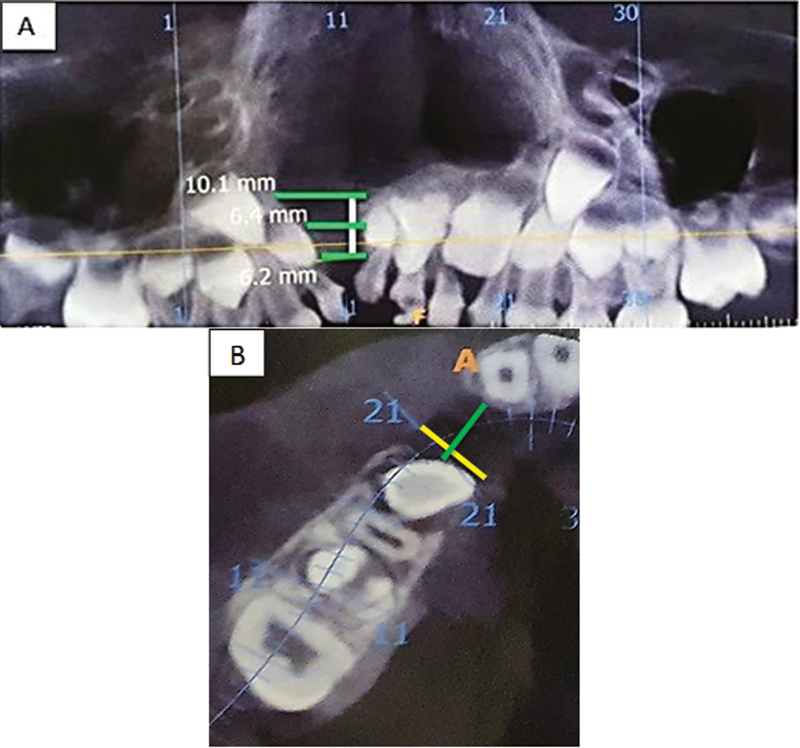
Bone volume assessment in the clefted site. ( **A** ) Orthopantomography show green line show different length levels of the clefted site. ( **B** ) CBCT show the diameter of the clefted site (yellow line). Green line show length of the clefted site. CBCT, cone beam computed tomography.

All child patients are clinically evaluated to determine the need for soft tissue bed before bone graft surgery, and the bone defect is graded into three categories: small (less than 1 cm ^3^ , seen in 9 patients), medium (1–3 cm ^3^ ), and severe (more than 3 cm ^3^ , detected in 26 patients). CBCT images were evaluated using an in vivo dental image analysis software, with three anatomical landmarks for each patient gathered, including facial width, facial height, and facial-palatal length. This is the first volume estimated of the cleft. All measurements were taken by a single rater with the same center and device at three different times. Pre- and postoperative CBCT images are taken to evaluate the volume of the bony defect before surgery as well as the bone healing process postoperatively ( Figs. 1 and 2 ).

A special case sheet was prepared by the authors to fit all the assessment information, starting with demographical information and clinical and radiographic examination notes. The case sheet also includes a detailed description of the surgery, as well as its complications and a minimum 1-year follow-up.

#### Multidisciplinary Team Clinic for Cleft Lip and Palate

All patients were assigned to the MDT CLP, and final discussions of case management, type of surgery, and dating of operations are performed through the committee decision.

#### Surgery

All patients are operated on by the same surgeon in both soft tissue and bone grafting surgeries. Soft tissue surgery was endured at least 2 months prior to bone grafting by the same maxillofacial surgeon under general anesthesia. Soft tissue surgery will provide the soft tissue bed to receive the bone graft material later on.

### Bone Graft Surgery

#### Surgical Procedure

**Fig. 3:**
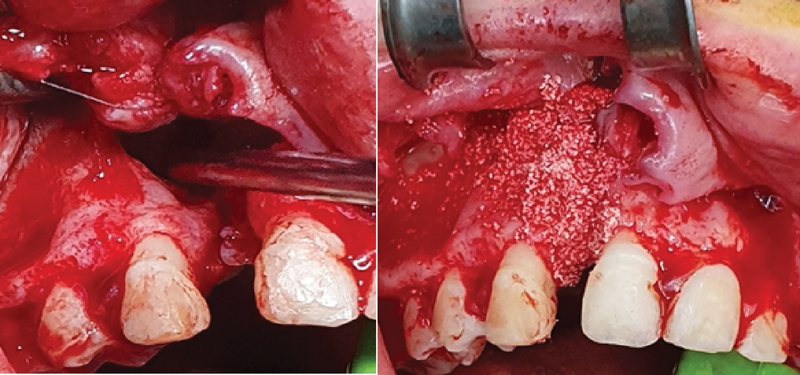
Alveolar cleft with soft tissue reflection and Osteon III bone substitute filling the defected bone area.

Standard surgical principles for AC grafting are followed: under general anesthesia, assist with the infiltration of adrenaline as a vasoconstrictor under local anesthesia. Design the mucoperiosteal gingival flaps along the clefted margin. Gentle flap reflection in both lateral and medial directions extends posteriorly to the area of the first molar and back-cuts up to the buccal sulcus to perform free movement of the mucoperiosteal flap posteriorly; keep adequate soft tissue cover of the grafted material ( Fig. 3 ). Separate the soft tissue pocket in a nasal-palatal direction along the grafted edges with complete exposure of all bony clefts in 3D margins.

After full exposure of the clefted site, bone grafted materials (Osteon III) can be deposited in the area with absolute perfect adaptation. The operator adapt the bone substitute into the cleft site by applying gentle pressure.

Finally, a tension-free flap closure with water-proof criteria is maintained to accurately close the grafted materials. A broad-spectrum cephalosporin antibiotic was given for 5 days via the oral route. On the next postoperative day, patients are discharged home.

### Bone Graft Material Source

**Fig. 4:**
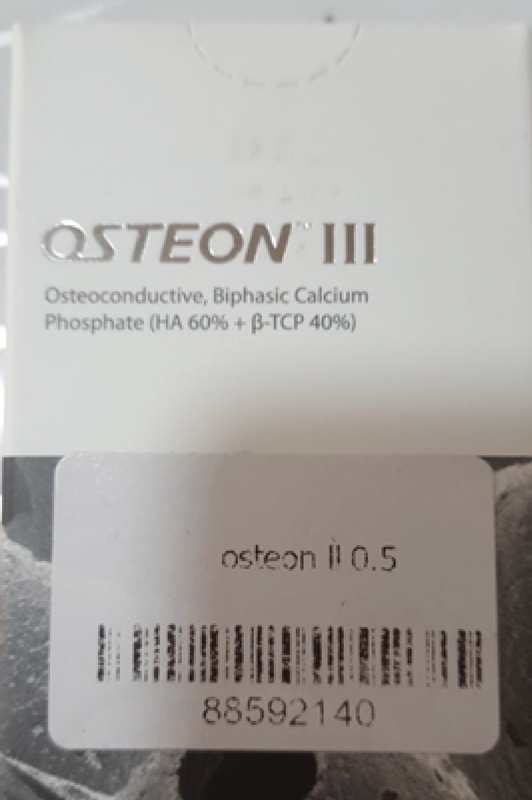
Osteon III bone substitute as a reconstruction material.

Osteon III is an artificial bone material. It is a widely used allogeneic material for reconstruction of bone defect consisting of HA and TCP. In this study, 0.5 mg vials are only mixed initially with few drops of distilled water to perform soft wet homogenous mixture adapted very well to the alveolar defect site. The numbers of Osteon III vials used in each surgery are different according to the size of bone defect ( Fig. 4 ).

### Bone Defect Volume Evaluation

**Fig. 5:**
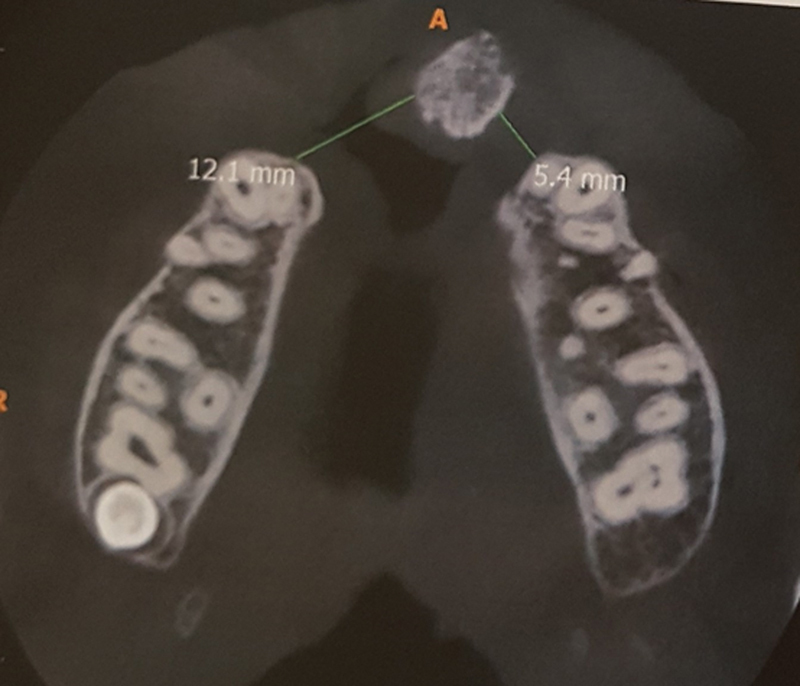
Bone volume estimation measurements of alveolus cleft by CBCT, 6-month postoperatively in the follow-up period. CBCT, cone beam computerized tomography.

CBCT is used for three times; preoperatively for first time to detect the correct volume of alveolus defect (V1); then to re-evaluate the bone healing process (volume of bone defect) after 3 months (V2); and 6 months later ( Fig. 5 ) to assess the proper amount of bone formed closing the defect (V3) postsurgery.

### Statistical Analysis

Descriptive study analysis of the sample includes various parameters such as AC grade (small, medium, large), bone defect volume (V1) at first radiographic assessment with CBCT, bone defect volume at second radiographic assessment with CBCT after 3 months (amount of unhealed defect, V2), and bone defect volume at second radiographic assessment with CBCT after 6 months (amount of bone formed closing the defect, V3). By using the SPSS software program, analysis of the data was performed.

The means and standard deviations are utilized. A paired sample *t* -test is employed for comparison as well as to evaluate *p* -value significancy ( *p* ≤ 0.001).

## Results

The total number of participants was 55, whose ages ranged from 6 to 13 years (mean: 9.2545 years). Twenty-five patients are male, and the other 30 are female. Cleft degree was classified as small (9 cases/16.3%), medium (20 cases/36.3%), and large (26 cases/47.2%).

Table 1 displays descriptive analysis for the sample, which includes bone volume of the clefted site divided into three steps: volume of bone defect at first-step examination (mean: 18.1091 mm ^3^ ), volume 2 recorded after 3 months, which resembles the amount of unhealed defect (mean: 0.5109 mm ^3^ ), and final bone volume assessment after 6 months (amount of bone formed closing the defect: volume 3; 22.5455 mm ^3^ ).

**Table 1:** Descriptive analysis of patient's sample (bone volume in three steps of the study)

Parameters	a V1 (cm ^3^ )	b V2 (cm ^3^ )	c V3 (cm ^3^ )
Cases, total number	55	55	55
Mean	18.1091	0.5109	22.5455
Standard deviation	7.54167	0.74004	9.12631

a V1 = bone defect volume at first radiographical assessment with cone beam computerized tomography (CBCT).

b V2 = bone defect volume at second radiographical assessment with CBCT after 3 months/amount of unhealed defect.

c V3 = bone defect volume at second radiographical assessment with CBCT after 6 months/amount of bone formed closing the defect

### Statistical Analysis of Bone Volume (V1, V2, and V3)

#### Relation between V1 Preoperatively, V2 and V3 Postoperatively

Assessment of bone defect volume is done in the clefted site in three stages: preoperatively (V1), postoperatively after 3 months (V2), and at 6 months (V3). Healing process evaluation with the bone substitute reconstruction of the alveolus cleft is described in Table 2 : *t* -test significant difference and correlation between V1, V2, and V3. *p-* Value ( *p* ≤ 0.01) was used statistically to reflect the significance difference between parameters. Table 2 shows the mean and standard deviation between V1 and V2, with a significant correlation (0.005) between them reflecting the change in bone volume after 3 months. The same assessment was performed between V2 and V3, with a significant *p* -value in the *t* -test paired sample also presented (0.001).

**Table 2:** Paired sample statistics and correlation ( *t* -test for V1/V2 and V2/V3)

Paired samples		Mean	Standard deviation	Correlation	Highly significant a, b
Pair 1	V1	18.1091	7.54167	0.374	**0.005**
	V2	0.5109	0.74004		
Pair 2	V2	0.5109	0.74004	0.439	**0.001**
	V3	22.5455	9.12631		

Note: V1 = bone defect volume at first radiographical assessment with CBCT. V2 = bone defect volume at second radiographical assessment with CBCT after 3 months/amount of unhealed defect. V3 = bone defect volume at second radiographical assessment with CBCT after 6 months/amount of bone formed closing the defect. Statistically significant *p* -values are indicated in bold.

a *p-* Value considered significant at *p* ≤ 0.01.

b *p-* Value highly significant (0.005) result of *t-* test paired sample (V1/V2).

c *p-* Value highly significant (0.001) result of *t-* test paired sample (V2/V3).

#### Difference Rate between V1 Preoperatively, V2 and V3 Postoperatively

Table 3 emphasizes the difference rates between V1, V2, and V3, with consequence *p-* value significancy. Both show statistically significant differences in bone volume formation.

**Table 3:** Difference rates between V1 and V2, and between V2 and V3 with consequence *p-* value significancy

	Paired differences				*t*	df	Significance (two-tailed) abc Highly significant
	Mean	Standard deviation	95% Confidence interval of the difference				
			Lower	Upper			
Difference between V1 and V2	17.59818	7.29720	15.625	19.570	17.885	54	**0.000**
Difference between V2 and V3	22.03455	8.82679	24.42076	19.64833	18.513	54	**0.000**

Note: V1 = bone defect volume at first radiographical assessment with CBCT. V2 = bone defect volume at second radiographical assessment with CBCT after 3 months/amount of unhealed defect. V3 = bone defect volume at second radiographical assessment with CBCT after 6 months/amount of bone formed closing the defect. Statistically significant *p* -values are indicated in bold.

a *p-* Value considered significant at *p* ≤ 0.01

b *p-* Value highly significant (0.000) result of *t-* test paired sample (V1/V2).

c *p-* Value highly significant (0.000) result of *t-* test paired sample (V2/V3).

Concerning complication, three patients only express exposure of the grafted materials and lack of bridging in the site.

## Discussion

Abnormal growth and development or failed fusion of the fronto-nasal prominence will end with an AC. Several studies discussed that the ideal period for achieving bone grafting for ACs is during the mixed dentition stage. 15 In this article, we focus on secondary alveolar grafting in patients aged between 6 and 13 years because there are many controversies about the newly discovered synthetic materials due to the advancement of scientific knowledge. Age, gender, types, staging, and bone defect volume are discussed widely in articles. 5 7

Closure of the oro-nasal fistula, maintaining maxillary arch continuity to amplify dentition bone support, and maxillary bone segment stabilization are crucial for orthodontic treatment and others. For these reasons, bone grafting or repair in cleft patients is crucial and highly indicated to permit eruption of the teeth in the grafted site and the eruption of the canine without the use of orthodontic traction.

Grafting materials are different and range over a wide variety with various body responses; bone substitutes have been used in recent years because of limited bone retrieval despite the fact that autologous bone is the ideal graft material as stated in many published articles. 16

Multiple bone-augmentation materials are well known. Each method has pros and cons that can vary according to bone volume deficiency. Iliac crest grafting (autogenous) has proved to be the gold standard site and material for bone grafting in comparison with alloplastic synthetic materials (bone substitutes). Alveolus grafting requires high accuracy and skill in surgeries because there is a high possibility that graft resorption or notching in the alveolar bone will occur. This complication occurs due to a soft tissue cover deficiency. 17 From another point of view, iliac crest surgeries can be complicated by persistent pain percentages ranging from 0 to 49%, the rate of sensory disturbance of the lateral femoral cutaneous nerve ranging from 2.9 to 27%, and 4.3 to 17% for permanent functional disorders, as stated by Almaiman et al in their published article from 2013. 18

In a published article by de Rezende Barbosa et al, it was stated that CBCT is reliable for the volumetric assessment of AC defects when using methods of area determination in axial reconstruction and segmentation with 3D rendering of the volume. Contingent on this advanced technology, in this study, bone volume was checked three times, as stated previously, to determine the exact bone volume defect and/or amount of bone formed. 19

The following principles were adopted: concerning the best time for AC grafting before eruption of the canine tooth in mixed dentition to provide good health conditions for adjacent teeth, confirm the accurate eruption path for the canine, and reduce facial growth discrepancy. 20 In this study, one of the most important advantages is that less surgical morbidity is associated with uneventful canine eruptions. In spite of the fact that the authors did not include canine eruption as a variable in this study, most patients were surprised by canine eruption at the clefted site subsequently.

In this article, the authors focus on the response of bone substitute (Osteon III) as a grafting material on the basis of bone volume change presurgery, 3 months after surgery, and 6 months postsurgery. This period will give clue to the bone healing process, both clinically and radiographically.

In V1, the authors use the bone defect volume; in V2, the authors also use the amount of defect volume as the bone substitute is condensed on the bone at the site of the defect, filling all the defect at the time of surgery. In the third month, the osteon has not yet resorbed, and we think that measuring the rest of the defect gives a better view of the site. In the third measure, after 6 months, it is supposed that the bone substitute will change to bone, so we can measure the bone volume accordingly.

All cases chosen are unilateral clefts, in similarity to articles published by Du et al in the same year claiming that most cases are unilateral and specifically left-sided. 21

Significant differences in the bone volume are estimated with good bone healing and can be manifested in the study sample through CBCT multiple evaluation. At a later stage, bone substitutes can still produce good results and are considered as successful as alveolar bone grafting. After the complete healing process of the grafted material has been approved, all patients are followed to the next step.

Allograft materials are currently used in implant dentistry as a means of sinus lifting or socket preservation, as well as an attractive alternative, according to different published articles. They have superior characteristics in donor site morbidity avoidance and various osteoconductive capabilities. 22 They have the following advantages: no need for a donor site, abundant supply, little risk of infection, and easier manipulation. The “ideal alloplastic bone substitutes are biologically stable and maintain their volume with cell infiltration and remodeling processes.” 21

In 2009, researchers such as Goudy et al 23 advocated the hope of using allogeneic materials. “The revision rate in our series is similar to others, and we did not find that the use of DBM changed the results in our patients. It is our hope that with bone-inductive substances, we will find that even fewer revision surgeries are necessary.” 23

In a review article published in 2011 by Cho-Lee et al, analysis of secondary alveolar grafting highlighted the fact that it is preferable to use a corticocancellous block of iliac bone in combination with bone chips. They do not prefer allogeneic materials in their protocol. 24

Jamjoom and Cohen in 2020 conclude in their published review article that the use of bone substitutes has increased widely in the last few years because of their ease of manipulation, low morbidity, excellent productivity, and good functioning as scaffolds with growth factors. In 50 of the cases where fractures occurred after bone harvesting, the area harvested was the anterior iliac crest. This complication appears to be caused by a combination of the harvest site, residual bone thickness, surgical approach, and patient age and gender. 25

Allografts (obtained from genetically similar members of the same species), xenografts (obtained from other species), and alloplasts are just a few of the bone substitutes currently on the market (of synthetic origin). Bone substitutes should be biocompatible, fully resorbable, not antigenic or carcinogenic, cheap, and not transmit diseases if they are to be used in place of natural bone. In addition to filling the void, they should be composed of particles of the same size and resorb at the same rate as the human bone. 26

In order to be an effective bone substitute, a material must possess two key characteristics: the scaffold ability for osteoconductivity and good integration qualities appropriate for growing cells, blood vessels, and growth factors for osteoinductivity. Bone substitutes should match the biomechanical properties of natural bone, be biocompatible with the body, and degrade over time. Bone substitutes that meet these criteria abound on the market. Take Osteon III bone substitute as an example.

Osteon III is considered the third generation of alloplastic bone substitute, composed of hydroxyl apatite and TCP (60–40%). It has the superior advantages to other types as it is characterized by higher porosity, interconnection, and crystallinity.

In an animal (rabbit) study, 25 the focus was on osteoblast and osteoclast activity, and it was agreed that the material exhibited excellent scaffold properties in comparison to a conventional alloplastic bone graft material. For its superior feature, we use it in the present study as an alternative to autogenous bone grafting materials, avoiding all complications that can occur in bone harvesting from the donor site. Bone volume healing processes with a significant difference can reflect the safety of this material.

The rationale for using this Osteon III material is specifically related to the fact that it is widely used in bone tissue engineering and that it is a mixture of two important substances (HA crystals and calcium phosphate). Many articles focus on the use of these materials alone or in combination with other materials such as collagen and chitosan to develop new scaffold strategies. 27 28 29 30 31 32 33 34

Clinical studies by researchers such as Honma et al, 35 Trindade et al, 36 Tan et al, 37 and Enemark et al 38 discuss the bone-bridge volume in the grafted site with various patient samples (15, 65, 100, and 95 patients, respectively) and different periods of observation ranging from 3 months to 1 year, and to 4 years and 5 years. They concluded that in a period of 1 year, the bone volume change can be variable, which can be related to teeth eruption in the clefted site and orthodontic treatment. For a long-term follow-up (4–5 years), the authors indicate that the normal bone height can be detected. In this article, patients were followed for a period ranging from 6 months to 4 years. Although the assessment was based on clinical rather than radiographic evaluation, there were no unpleasant complications recorded in the follow-up period. On the basis of reviews of different articles in this study, the authors use bone substitutes as a graft material to reconstruct the AC. As highlighted in the “Results” section, the use of bone substitute gave a significant difference (increase) in bone volume with the 6-month follow-up periods.

The authors here agree that a long period of observation time for bone grafted in the nasoalveolar cleft site may, to some extent, be helpful for estimation of surgical outcome.

In spite of that, autologous cancellous grafting materials are most widely accepted as treatment protocols in patients aged between 6 and 13 years, but alloplastic bone substitutes can also be used because of limited bone retrieval. Osteon III bone substitute can be used as an alternative material to reconstruct the alveolar bone cleft site. More research is needed to assess the combination between osteoinduction activity displayed in allogeneic demineralized bone and osteoconduction activity-revealed mineralized bone to improve the bone healing process in the clefted site. Table 4 highlights the pros and cons of different raft materials. 39

**Table 4:** Advantages and disadvantages of the most commonly used three types of bone grafts 39

Bone graft	Advantages	Disadvantages
Autograft	Optimal osteogenic, osteoinductive, and osteoconductive properties; gold standard for bone grafting; without the risks of immunogenicity and disease transmission	Pain and morbidity in the donor site, limited quantity and availability, need for further surgery, hematoma, infection, the need for general sedation or anesthesia, longer operative time, and blood loss
Allograft	Osteoinductive and osteoconductive properties, without donor-site morbidity, possible with local anesthesia, high availability, easy handling	Lack of osteogenic properties, potential antigenic response, and disease transmission, variable osteoinductivity, limited supply, loss of biologic and mechanical properties due to its processing, nonavailability worldwide due to religious and financial concerns and increased cost
Xenografts	Osteoinductive and osteoconductive properties, low cost, high availability	Lack of osteogenic properties, the risk of immunogenicity and transmission of infectious and zoonotic diseases, poor outcome

However, there are drawbacks and risks associated with using bone substitute as a graft material. Depending on material characteristics like bioabsorption and, in some cases, union continuity, this can be a challenging process. Osteoconductivity alone causes mechanical defenselessness. According to a review published by Sohn et al in 2019, it is difficult to choose the best bone substitute because no material can fully replace autogenous bone. Factors such as the size of the bone defect, the size and shape of the graft material, its biomechanical properties, its ease of manipulation, its cost, ethical issues, its biological properties, and complications must all be taken into account when assessing the biological and mechanical characteristics in each clinical situation. 40

The healing process and, ultimately, the graft's success and integration are profoundly influenced by its 3D position. 41 As a result, the use of iliac crest bone graft versus synthetic grafts (-TCP/HA) is highly controversial due to the lower fusion rates of the former and the higher risks of graft fragmentation, settling, and instrumentation problems of the latter. 42

In a mini-review done by Valtanen et al in 2020, they emphasize the segmental defect treated by engineered synthetic bone substitutes with their limitations and risks, and conclude that “bone tissue engineering remains an exciting prospect for the treatment of large segmental bone defects; however, current clinical integration of engineered scaffolds remains low.” The authors of this mini-review believe that clinical application obstacles lie in the lack of concomitant vascularization of these scaffolds. 43

Despite the fact that synthetic grafting materials eliminate these risks, they do not transfer osteoinductive or osteogenic elements to the host site, as recommended by clinicians, and a 2002 article by Betz clarifies that allograft is the next best alternative for autograft with minor immunogenic rejection and the risk of disease transmission as unresolved issues. Composite grafts, which combine the benefits of autografts and allografts, are an option for patients who want the best of both worlds. To promote cell infiltration and bone formation, such a graft may include both a synthetic scaffold and biologic elements. 44

Limitations in this study include: increasing the sample size is preferable; inclusion of different cases is needed in relation to orthodontic treatment and canine relation eruption; and a comparison study between different materials also needs to be conducted.

Bone graft and substitute materials which are either in the form of particulate or blocks are available in wide variety with wide controversy to be used as a graft material besides being mostly used in dentistry to regenerate the missing hard tissue structures. Cleft alveolus is a well-known congenital anomaly which can be seen in Iraq with increased demand to find a new efficient dental grafting material. In fact, bone substitutes have a wide variety of dental uses and demonstrated its value in implant surgery through applications such direct sinus lifting, nerve transposition, and maintenance of the implant socket. From this point of view, it is important to study the role of bone substitute as graft materials in alveolus cleft patients. The bone graft materials should act as a structural framework for osteo-regenerative processes that only satisfy the osteoconductivity criteria a long side resists the host. In this article, the authors agreed the idea that bone substitute can to some extent replace the gold standard autogenous bone grafting materials. However, despite that, multiple advanced research studies are needed to solve the controversy.
